# Two-Year Follow-Up after Endovenous Closure with Short-Chain Cyanoacrylate versus Laser Ablation in Venous Insufficiency

**DOI:** 10.3390/jcm10040628

**Published:** 2021-02-07

**Authors:** Justyna Wilczko, Cezary Szary, Dominika Plucinska, Tomasz Grzela

**Affiliations:** 1 Clinic of Phlebology, 02-034 Warsaw, Poland; justyna.wilczko@klinikaflebologii.pl (J.W.); cezary.szary@klinikaflebologii.pl (C.S.); dominika.plucinska@klinikaflebologii.pl (D.P.); 2 Diagnostic Imaging Center MRI & CT, Center of Sport Medicine, 02-034 Warsaw, Poland; 3 Department of Histology & Embryology, Medical University of Warsaw, 02-004 Warsaw, Poland

**Keywords:** chronic venous disease, cyanoacrylate, endovenous procedure, ELVeS, laser thermoablation, non-thermal ablation, recanalization, recurrence, VenaBlock

## Abstract

Background: The current treatment of venous disease is focused on the minimally invasive exclusion of the affected vein. Besides widely used thermal ablation, chemical ablation with cyanoacrylate, reported as safe and highly effective, has been gaining increasing interest. Patients and methods: In the current report, we present data from a two-year observation in 89 patients (61 female/28 male, mean age 44.3 ± 13.5) suffering from venous insufficiency (C2–C4), treated either using short-chain cyanoacrylate, the VenaBlock system (*n* = 43) or laser thermoablation with ELVeS 1470 (*n* = 46). The assessment comprised the occurrence of venous disease-related symptoms and the ultrasound examination of the leg venous system. Results: The frequency of recanalization after 2 years from the VenaBlock procedure was significantly higher than after laser treatment (37.2 vs. 8.7%). Apart from recanalization, in some individuals from both groups, the symptoms of recurrence and/or disease progression, including the development of insufficiency in other veins of treated or contralateral legs (9.3 vs. 15.2% and 9.3 vs. 17.4%, respectively), were observed. Unexpectedly, the general prevalence of the disease progression did not differ significantly between the VenaBlock and ELVeS groups (44.2 vs. 34.8%, respectively). Conclusions: Despite the higher recanalization rate of VenaBlock compared to ELVeS, the overall effectiveness of cyanoacrylate and laser thermoablation after two years was similar. Therefore, both methods similarly failed to prevent recurrence and disease progression, which seem to be method-independent.

## 1. Introduction

Chronic venous disease (CVD) is a common clinical problem, especially in industrialized countries. It is characterized by a broad spectrum of symptoms, whichsignificantly impair the patient’s life quality, with serious complications at more advanced stages [1,2]. Due to the chronic character of the disease and the still increasing prevalence, CVD emerges as an important health and social problem [2,3]. The treatment of CVD is usually focused on the elimination of reflux in superficial veins. Apart from standard ligation and stripping surgery, currently the most preferred approach comprises minimally invasive endovascular methods, including both the thermal and non-thermal ablation of insufficient venous trunk [4,5]. Thermal methods include radiofrequency- and laser-based ablation, whereas non-thermal techniques comprise foam and liquid sclerotherapy, mechano-chemical obliteration, or the recently introduced endovenous application of cyanoacrylate [6,7,8]. The latter was announced as highly effective in the treatment of truncal insufficiency of large superficial veins—mainly great saphenous (GSV) and anterior accessory saphenous veins (AASV) [8,9,10]. High occlusion rate reported in the first VeClose trial has encouraged several manufacturers to further develop this technique [9]. Hence, in addition to slowly polymerizing, high-viscosity cyanoacrylate, VenaSeal (Medtronic, Minneapolis, MN, USA), used in the VeClose study, recently the short-chain obliterating agent named VenaBlock (Invamed Saglic Ilac A.S., Ankara, Turkey), which is characterized by its low viscosity and fast polymerization, was introduced to the market [10,11,12,13].

The modified composition and the rapid binding of this form of cyanoacrylate demanded significant alterations in the technique of its application, which in turn could affect the safety and effectiveness of this method, especially when considering the risk of recanalization. Indeed, our previous report from the single-center prospective ESVETIS (Efficacy and safety of VenaBlock vs. ELVeS systems in the treatment of truncal insufficiency in superficial veins of lower limbs) study has shown that at a 6-month follow-up, the recanalization concerned 9.7% of cyanoacrylate-treated veins [14]. Although the recanalization in patients from the control group subjected to endovenous laser thermoablation with ELVeS 1470 nm (Leonardo Dual 45, Biolitec/CeramOptec GmbH, Bonn, Germany) after 6 months was found in 2.2% of veins, this difference was non-significant. Both VenaBlock and ELVeS did not differ with regards to the occurrence of a serious adverse event or CVD-associated symptoms reduction. However, when analyzing the overall effectiveness of treatment, apart from the short-term risk of recanalization in the treated veins or the selected symptoms of disease, long-term outcomes and the features of disease progression are to also be examined. Therefore, the aim of the current study was the assessment of mid-term results in patients from the ESVETIS trial, two years after the treatment of saphenous vein insufficiency with cyanoacrylate or laser thermoablation.

## 2. Patients and Methods

The characteristics of the study group and methods relate to a previously published 6-month follow-up of the prospective interventional non-randomized ESVETIS trial [14]. Here we report a two-year follow-up of the same patients, with the exception of two individuals who were added to the original cohort after the submission of the aforementioned report.

In brief, 91 consecutive patients were allocated to study groups alternately (1:1), however, since two individuals from the VenaBlock group did not attend any control visit, they were retracted from further analysis. Hence, for the final assessment, there remained 43 individuals in the VenaBlock group and 46 patients in the ELVeS group. The main inclusion criteria for this trial were age above 18, GSV, AASV or small saphenous vein (SSV) reflux duration above 1 sec at color-duplex ultrasound (CDU) examination, initial trunk diameter in standing position >4 mm and <10 mm, and clinical stage between C2 and C4, according to clinical, etiological, anatomical and pathophysiological (CEAP) classification [15]. The main exclusion criteria for the ESVETIS study comprised previous interventions in the veins to be treated, C5 and C6 CEAP stages, primary or secondary deep vein disease, acute or subacute venous thrombosis, known allergy to cyanoacrylates, severe systemic disease, pregnancy or breastfeeding and active cancer or any oncologic treatment within the last 5 years. Neither patients nor investigators were blinded to the treatment, as it was impossible to effectively maintain them blinded due to substantial differences in both methods: the procedure itself, the use (or not) of tumescence and compression, and especially the characteristic hyperechogenic deposit of cyanoacrylate, visible in the ultrasound scan of the obliterated vein [16].

In the current research, the included patients were the ones who accomplished the previous 12-month observation in the ESVETIS trial and were willing to attend one additional control visit within 24 months after the procedure. All patients gave their informed written consent to participate in the trial. The basic protocol of the study and its amendment were reviewed and approved by the Local Bioethics Committee of the Medical Council (approval No. KB/1073/17).

As previously described, the further assessment comprised control visits after 1, 6 and 12 months from the procedure. The assessment included CDU examination of the treated limb with the morphometric evaluation of the obliterated vein in previously specified points, as well as the patient’s self-assessment of selected symptoms using a 10-point visual analogue scale (VAS) for pain/discomfort and our simplified non-validated CVD-oriented quality of life questionnaire, basically similar to VVSymQ [17]. However, due to unexpectedly poor results in the cyanoacrylate-treated group, at least when compared to other reports, we decided to extend the observation period to the next 12 months. Hence, patients were invited for an additional visit, with the detailed CDU examination of both limbs and careful revision of recent patient records.

It is noteworthy that in the 2-year assessment no adverse events were reported or found. Any deterioration in the occurrence and/or intensity of symptoms, or any CDU-based new pathological findings in the treated veins and in previously competent veins were recorded; these data were collected so as to assess any possible manifestation of disease progression and/or recurrence. Therefore, special attention was paid to the occurrence of any symptoms of recurrence or disease progression; more specifically, we recorded any recanalization of the treated vein (at any length), the possible progression of incompetence in other saphenous or trunk veins of the treated limb, or any new truncal reflux in the contralateral leg. Furthermore, to improve the accuracy of our data, we excluded any post-procedure treatment from the final evaluation. Thus, any necessity to use any further intervention with regards to the treated vein (e.g., foam sclerotherapy) was considered as treatment failure.

The analysis of collected data included descriptive statistics, comparative assessment between groups using the Mann–Whitney U test, as well as odds ratio (OR) with a 95% confidence interval (95% CI). The difference between analyzed parameters was considered statistically significant at *p* < 0.05.

## 3. Results

The brief clinical characteristics of both study groups were summarized in Table 1.

The comparative assessment performed within the first year after treatment in the ESVETIS study has shown that, except for a significantly shorter procedure with cyanoacrylate and significantly less pain/discomfort within 7 days after thermoablation, both methods were similar with regards to symptoms relief and the safety of treatment. The early results of the previously published ESVETIS trial [14] are briefly summarized in Table 2.

The ESVETIS study has revealed that the effectiveness of each procedure, expressed as the durability of vein occlusion and the risk of recanalization, varied among both methods at different time points. Six months after treatment, more veins were recanalized in cyanoacrylate than in the laser-treated group, but this difference did not reach statistical significance [14]. However, in subsequent assessment, the observed difference further increased and became statistically significant (Table 2).

According to the extended observation plan which pertains to this study, the additional visit took place in the vast majority of patients approximately 24 months from the original treatment, except three patients from the VenaBlock group and two individuals from the ELVeS group who attended the clinic after 20–21 months from the procedure. Since in all of those cases the treated veins were already recanalized (as recorded at 12-month assessment), in none of them the earlier visit influenced the clinical outcomes.

Our further clinical assessment after two years from the original procedure was mainly focused on the occurrence of any finding and symptoms of disease progression. The aforementioned vein recanalization was more frequent in cyanoacrylate-treated group and affected more than one-third of treated veins, compared to less than one-tenth among laser-obliterated vessels.

Apart from the recanalization of the treated vein, in patients from both groups, significant progression of vein incompetence was observed in other large vein/veins, both in the treated and the contralateral limb. Interestingly, although that phenomenon was more frequent in the ELVeS group, the observed difference did not appear statistically significant. The abovementioned findings from CDU examination were accompanied by a mild-to-moderate increase in symptoms, including leg heaviness and/or discomfort. However, their frequency or intensity was similar in both groups. It is noteworthy that besides the vein recanalization, none of the observed symptoms or features of recurrence or disease progression differed significantly between both groups (Table 3).

## 4. Discussion

Since the first approval of n-butyl cyanoacrylate for the treatment of truncal venous insufficiency in 2011, cyanoacrylate-mediated vein ablation was announced as a fast, painless and effective method in that indication [6,7,8,9,10,11,12,13,18,19,20]. The first two mentioned advantages result from no need for a time-consuming tumescent anesthesia. Thus, the duration of the vein ablation procedure using the first system which has been introduced into the market (VenaSeal) is similar or a bit shorter than that of laser thermoablation, mainly due to the slow polymerization (approximately 30–60 s) of the obliterating agent. However, in the case of the short-chain cyanoacrylate (VenaBlock system), the time necessary for the entire procedure is extremely short, usually below 10 min. This difference results mainly from the rapid polymerization (approx. 5 s) of this form of cyanoacrylate [10,11,12] that requires fast catheter traction during its application. Nevertheless, one can speculate that the latter may not guarantee the uniform application of the obliterating agent that is critical for the effective occlusion of the treated vein [10]. This condition may be particularly important for large-diameter veins [14,21]. In that case, the lack of tumescent anesthesia, although emphasized as the main benefit of cyanoacrylate-based procedures, does not prevent the presence of blood persisting in some segments of the vein after treatment. Since persisting blood will coagulate, subsequent fibrinolysis may result in recanalization, which may then be considered as the method-specific finding of treatment failure [10,18]. This assumption could be supported by the higher prevalence of recanalization in the VenaBlock group, observed even in the short-term assessment [14]. On the other hand, one has to note that reflux reappearance was not limited to this group exclusively. In fact, in a large randomized trial the diameter of the treated veins was recognized as the second, after CEAP clinical class, the most important predictor of recanalization also for laser thermoablation [21].

According to the results of several randomized trials and meta-analyses, the risk of recanalization depends, at least to some extent, on the method used for treatment [22,23,24]. In fact, a higher recanalization rate was documented in the case of foam sclerotherapy, whereas the lowest rate was found after surgical ligation and stripping [4,5,25]. Meanwhile, the same studies clearly indicated that recanalization is actually only one of several features of treatment failure [24,26]. It is noteworthy that this observation was confirmed also in our study. The ultrasound examination, performed two years after the procedure, apart from recanalization, in some patients from both groups has revealed the significant progression of CVD. The observed disease progression was manifested either by de novo occurrence, or by the increase in previous “borderline” reflux in other large veins in the treated and/or contralateral limb. Interestingly, in the ELVeS group, a less frequent recanalization but more frequent findings of ipsi- or contralateral progression were observed, whereas in the VenaBlock group, more frequent recanalization was accompanied by local progression on the same limb. All these findings were associated with an increase in leg heaviness and/or discomfort reported by the patients. Noticeably, although the patterns of recurrence slightly differed, the prevalence and general features of CVD progression in both groups were similar, and thus the global risk of recurrence appeared to be method-independent. Moreover, even the combination of various methods does not guarantee the avoidance of recurrence. As could be found in a recent report from the VeClose trial, despite an extremely high (94.4%) occlusion rate reported in the VenaSeal cyanoacrylate-treated group, the majority of patients received repetitive supplementary treatment, either using phlebectomy, sclerotherapy, or both [27,28]. This clearly demonstrates that saphenous vein occlusion is not always the equivalent of success in CVD treatment [23,24,25,27,28].

The results of our report are consistent with observations from several previous studies with mid-term follow-up [22,23,24,25,26]. Furthermore, a significantly higher rate of recanalization and recurrence has been demonstrated in long-term studies regarding varicose vein treatment [29]. The possible explanation for that phenomenon could be the underestimated role of alternative reflux sources in superficial veins, mainly of pelvic origin [30]. Therefore, major attention should be reserved for the proper identification of incompetent veins, both in the abdomino-pelvic and in the lower limb venous system, prior to any treatment [31,32].

In summary, despite the higher recanalization rate of short-chain cyanoacrylate (VenaBlock) compared to laser thermoablation (ELVeS) after two years from the procedure, the overall effectiveness of both methods was similar. Hence, both of them failed similarly to prevent recurrence or disease progression. In that respect, the selection of any particular method for the treatment of lower limb veins seems to be a non-decisional issue.

## Figures and Tables

**Table 1:** Baseline features of the two treated groups. Values in table represent mean ± standard deviation, or number (percent) in the group, respectively.

Parameter or Variable	VenaBlock (*n* = 43)	ELVeS (*n* = 46)
Age	46.1 ± 14.4	42.5 ± 11.2
Female/Male (%)	30/13 (69.8/30.2%)	31/15 (67.4/32.6%)
BMI	25.2 ± 3.6	23.9 ± 3.5
Comorbidities:		
- Mild arterial hypertension	8 (18.6%)	6 (13.0%)
- Allergic asthma	3 (7.0%)	5 (10.9%)
- Hashimoto disease	6 (13.9%)	4 (8.7%)
Previous treatment (in other veins):		
- Surgery	1 (2.3%)	2 (4.3%)
- Thermoablation	2 (4.6%)	1 (2.2%)
- Sclerotherapy	5 (11.6%)	8 (17.4%)
Saphenous junction diameter (mm)	7.4 ± 1.8	7.6 ± 1.5
Length of treated trunk (cm)	44.2 ± 11.1	42.0 ± 10.4
Mean QoL score before treatment	13.7 ± 6.5	14.9 ± 6.2
Clinical stage (CEAP):		
- C2	34 (79.1%)	32 (69.6%)
- C3	6 (13.9%)	10 (21.7%)
- C4	3 (7.0%)	4 (8.7%)
Treated veins:		
- GSV	36 (83.7%)	38 (82.6%)
- AASV	2 (4.6%)	4 (8.7%)
- SSV	5 (11.6%)	4 (8.7%)

Abbreviations used: BMI—body mass index; QoL—quality of life; CEAP—clinical, etiological, anatomical and pathophysiological classification; GSV—great saphenous vein; AASV—anterior accessory saphenous vein; SSV—small saphenous vein.

**Table 2:** Comparison of the results for both methods with regards to selected clinical features assessed in the ESVETIS study within 1 year after procedure. Values in the table represent the mean ± standard deviation, or patient number (percent) in the group, respectively. Statistically significant differences were marked with asterisks (* *p* < 0.05).

Procedural Features or Related Symptoms	VenaBlock (*n* = 43)	ELVeS (*n* = 46)	OR	95% CI
Pain during procedure (VAS)	3.5 ± 2.3	2.7 ± 2.0	1.9 ^(1)^	0.5–7.2
Procedure duration (in minutes)	7.1 ± 4.6	17.0 ± 4.2 *	0.1 ^(2)^	0.04–0.3 *
Pain/discomfort within 7 days after procedure (VAS)	4.3 ± 2.4	2.9 ± 2.4 *	3.4 ^(3)^	1.0–10.8 *
Adverse events within 1 month after procedure	7 (16.3%)	6 (13.0%)	1.3	0.4–4.2
QoL score improvement within 1 year after procedure	35 (81.4%)	34 (73.9%)	1.5	0.6–4.0
Vein recanalization:				
- within 6 month	6 (13.9%)	1 (2.2%)	7.3	0.8–63.3
- within 12 months	13 (30.2%)	3 (6.5%)	6.2	1.6–23.7 *

Legend: OR—odds ratio; CI—confidence interval; VAS—visual analogue scale; QoL—quality of life; OR were calculated for VenaBlock vs. ELVeS as a control. ^(1)^ and ^(3)^ were assessed for the occurrence of pain >5; ^(2)^ was assessed for procedure duration > 10 min.

**Table 3:** Summary of the main features and symptoms of disease progression assessed 2 years after procedure. Values given in table represent the mean ± standard deviation, or patient number (percent) in the group, respectively. Statistically significant differences were marked with asterisks (* *p* < 0.05).

Feature or Symptom	VenaBlock (*n* = 43)	ELVeS (*n* = 46)
Time from the procedure (months)	23.6 ± 1.1	23.9 ± 0.9
Recanalization of the treated saphenous trunk	16 (37.2%)	4 (8.7%) *
Progression of incompetence in other trunks in the treated limb	4 (9.3%)	7 (15.2%)
Disease progression in the contralateral limb	4 (9.3%)	8 (17.4%)
Any symptoms of recurrence	19 (44.2%)	16 (34.8%)
QoL change (vs QoL 1 year after procedure):		
- score improvement	7 (16.3%)	11 (23.9%)
- score not changed	21 (48.8%)	22 (47.8%)
- score worsening	15 (34.9%)	13 (28.3%)

Legend: QoL—quality of life.

## References

[1] Eberhardt R.T., Raffetto J.D. (2014). Chronic venous insufficiency. Circulation.

[2] Davies A.H. (2019). The seriousness of chronic venous disease: A review of real-world evidence. Adv. Ther..

[3] Raffetto J.D., Mannello F. (2014). Pathophysiology of chronic venous disease. Int. Angiol..

[4] Lawaetz M., Serup J., Lawaetz B., Bjoern L., Blemings A., Eklof B., Rasmussen L. (2017). Comparison of endovenous ablation techniques, foam sclerotherapy and surgical stripping for great saphenous varicose veins. Extended 5-year follow-up of a RCT. Int. Angiol..

[5] Van der Velden S.K., Biemans A.A., De Maeseneer M.G., Kockaert M.A., Cuypers P.W., Hollestein L.M., Neumann H.A., Nijsten T., van den Bos R.R. (2015). Five-year results of a randomized clinical trial of conventional surgery, endovenous laser ablation and ultrasound-guided foam sclerotherapy in patients with great saphenous varicose veins. Br. J. Surg..

[6] Almeida J.I., Javier J.J., Mackay E., Bautista C., Proebstle T.M. (2013). First human use of cyanoacrylate adhesive for treatment of saphenous vein incompetence. J. Vasc. Surg. Venous Lymphat. Disord..

[7] Vos C.G., Ünlü Ç., Bosma J., van Vlijmen C.J., de Nie A.J., Schreve M.A. (2017). A systematic review and meta-analysis of two novel techniques of nonthermal endovenous ablation of the great saphenous vein. J. Vasc. Surg. Venous Lymphat. Disord..

[8] Gibson K., Ferris B. (2017). Cyanoacrylate closure of incompetent great, small and accessory saphenous veins without the use of post-procedure compression: Initial outcomes of a post-market evaluation of the VenaSeal System (the WAVES Study). Vascular.

[9] Morrison N., Gibson K., Vasquez M., Weiss R., Cher D., Madsen M., Jones A. (2017). VeClose trial 12-month outcomes of cyanoacrylate closure versus radiofrequency ablation for incompetent great saphenous veins. J. Vasc. Surg. Venous Lymphat. Disord..

[10] Parsi K., Roberts S., Kang M., Benson S., Baker L., Berman I., Bester L.J., Connor D.E., Dinnen P., Grace J. (2020). Cyanoacrylate closure for peripheral veins: Consensus document of the Australasian College of Phlebology. Phlebology.

[11] Bozkurt A.K., Yılmaz M.F. (2016). A prospective comparison of a new cyanoacrylate glue and laser ablation for the treatment of venous insufficiency. Phlebology.

[12] Yasim A., Eroglu E., Bozoglan O., Mese B., Acipayam M., Kara H. (2017). A new non-tumescent endovenous ablation method for varicose vein treatment: Early results of N-butyl cyanoacrylate (VariClose^®^). Phlebology.

[13] García-Carpintero E., Carmona M., Chalco-Orrego J.P., González-Enríquez J., Imaz-Iglesia I. (2020). Systematic review and meta-analysis of endovenous cyanoacrylate adhesive ablation for incompetent saphenous veins. J. Vasc. Surg. Venous Lymphat. Disord..

[14] Wilczko J., Szary C., Plucinska D., Grzela T. (2019). A comparison of the safety and efficacy of the VenaBlock cyanoacrylate-based endovenous system versus 1470 nm endovascular biradial laser in the treatment of truncal insufficiency of superficial veins: Six-month outcomes of the ESVETIS observational study. Phlebol. Rev..

[15] Lurie F., Passman M., Meisner M., Dalsing M., Masuda E., Welch H., Bush R.L., Blebea J., Carpentier P.H., De Maeseneer M. (2020). The 2020 update of the CEAP classification system and reporting standards. J. Vasc. Surg. Venous Lymphat. Disord..

[16] McGuinness B., Elias F., Ali K.P., Ahmad M.S., Namburi J., Chan B., Szalay D., Rapanos T. (2019). A comparison of duplex ultrasound findings after cyanoacrylate embolization versus endovenous laser ablation of the great saphenous vein. J. Vasc. Surg. Venous Lymphat. Disord..

[17] Paty J., Turner-Bowker D.M., Elash C.A., Wright D. (2016). The VVSymQ^®^ instrument: Use of a new patient-reported outcome measure for assessment of varicose vein symptoms. Phlebology.

[18] Dimech A.P., Cassar K. (2020). Efficacy of cyanoacrylate glue ablation of primary truncal varicose veins compared to existing endovenous techniques: A systematic review of the literature. Surg. J. (NY).

[19] Kolluri R., Chung J., Kim S., Nath N., Bhalla B.B., Jain T., Zygmunt J., Davies A. (2020). Network meta-analysis to compare VenaSeal with other superficial venous therapies for chronic venous insufficiency. J. Vasc. Surg. Venous Lymphat. Disord..

[20] Çalık E.S., Arslan Ü., Erkut B. (2019). Ablation therapy with cyanoacrylate glue and laser for refluxing great saphenous veins—A prospective randomised study. Vasa.

[21] Van der Velden S.K., Lawaetz M., De Maeseneer M.G., Hollestein L., Nijsten T., van den Bos R.R., Members of the Predictors of Endovenous Thermal Ablation Group (2016). Predictors of recanalization of the great saphenous vein in randomized controlled trials 1 year after endovenous thermal ablation. Eur. J. Vasc. Endovasc. Surg..

[22] Gauw S.A., Lawson J.A., van Vlijmen-van Keulen C.J., Pronk P., Gaastra M.T., Mooij M.C. (2016). Five-year follow-up of a randomized, controlled trial comparing saphenofemoral ligation and stripping of the great saphenous vein with endovenous laser ablation (980 nm) using local tumescent anesthesia. J. Vasc. Surg..

[23] O’Donnell T.F., Balk E.M., Dermody M., Tangney E., Iafrati M.D. (2016). Recurrence of varicose veins after endovenous ablation of the great saphenous vein in randomized trials. J. Vasc. Surg. Venous Lymphat. Disord..

[24] Rass K., Frings N., Glowacki P., Gräber S., Tilgen W., Vogt T. (2015). Same site recurrence is more frequent after endovenous laser ablation compared with high ligation and stripping of the great saphenous vein: 5 year results of a randomized clinical trial (RELACS Study). Eur. J. Vasc. Endovasc. Surg..

[25] Rasmussen L., Lawaetz M., Serup J., Bjoern L., Vennits B., Blemings A., Eklof B. (2013). Randomized clinical trial comparing endovenous laser ablation, radiofrequency ablation, foam sclerotherapy, and surgical stripping for great saphenous varicose veins with 3-year follow-up. J. Vasc. Surg. Venous Lymphat. Disord..

[26] Rasmussen L., Lawaetz M., Bjoern L., Blemings A., Eklof B. (2013). Randomized clinical trial comparing endovenous laser ablation and stripping of the great saphenous vein with clinical and duplex outcome after 5 years. J. Vasc. Surg..

[27] Morrison N., Kolluri R., Vasquez M., Madsen M., Jones A., Gibson K. (2019). Comparison of cyanoacrylate closure and radiofrequency ablation for the treatment of incompetent great saphenous veins: 36-month outcomes of the VeClose randomized controlled trial. Phlebology.

[28] Morrison N., Gibson K., Vasquez M., Weiss R., Jones A. (2020). Five-year extension study of patients from a randomized clinical trial (VeClose) comparing cyanoacrylate closure versus radiofrequency ablation for the treatment of incompetent great saphenous veins. J. Vasc Surg. Venous Lymphat. Disord..

[29] Cavezzi A. (2020). Medicine and phlebolymphology: Time to change?. J. Clin. Med..

[30] Szary C., Wilczko J., Plucinska D., Pachuta A., Napierala M., Bodziony A., Zawadzki M., Leszczynski J., Galazka Z., Grzela T. (2021). The analysis of the selected morphological and hemodynamic parameters of venous system and their presumable impact on the risk of recurrence after varicose vein treatment. J. Clin. Med..

[31] Ratnam L.A., Marsh P., Holdstock J.M., Harrison C.S., Hussain F.F., Whiteley M.S., Lopez A. (2008). Pelvic vein embolisation in the management of varicose veins. Cardiovasc. Interv. Radiol..

[32] Hartung O. (2015). Embolization is essential in the treatment of leg varicosities due to pelvic venous insufficiency. Phlebology.

